# Multidisciplinary Interventions in Motor Neuron Disease

**DOI:** 10.1155/2014/435164

**Published:** 2014-11-18

**Authors:** U. E. Williams, E. E. Philip-Ephraim, S. K. Oparah

**Affiliations:** Internal Medicine Department, University of Calabar, Calabar, Cross River State 540242, Nigeria

## Abstract

Motor neuron disease is a neurodegenerative disease characterized by loss of upper motor neuron in the motor cortex and lower motor neurons in the brain stem and spinal cord. Death occurs 2–4 years after the onset of the disease. A complex interplay of cellular processes such as mitochondrial dysfunction, oxidative stress, excitotoxicity, and impaired axonal transport are proposed pathogenetic processes underlying neuronal cell loss. Currently evidence exists for the use of riluzole as a disease modifying drug; multidisciplinary team care approach to patient management; noninvasive ventilation for respiratory management; botulinum toxin B for sialorrhoea treatment; palliative care throughout the course of the disease; and Modafinil use for fatigue treatment. Further research is needed in management of dysphagia, bronchial secretion, pseudobulbar affect, spasticity, cramps, insomnia, cognitive impairment, and communication in motor neuron disease.

## 1. Background

Motor neuron disease (MND) also referred to as amyotrophic lateral sclerosis (ALS) is a fatal neurodegenerative condition with an annual incidence of about 1.5 per 100,000 [1] and a United Kingdom (UK) prevalence of 4–6/100,000 [2]. There is a slight male preponderance with a male to female ratio of 3 : 2. It could occur at any age but the peak age of occurrence is between 50 and 75 years [3].

Multiple genetic and environmental factors interact resulting in loss of the upper motor neuron in the motor cortex and the lower motor neurons cell bodies in the brain stem and spinal cord [4, 5]. Pattern of onset could be spinal, truncal, or bulbar. The clinical features of MND include limb weakness, respiratory impairment, dysphagia, fatigue, sleep disorders, pain, psychosocial distress, communication deficits, cognitive impairment, and spasticity. Death occurs secondary to respiratory failure 2 to 4 years after disease onset on average; however survival of patients up to a decade has been reported [6]. There is currently no cure for MND; hence management is focused on symptomatic treatment, rehabilitative care, and palliative care. The disease exerts a huge psychological and economic burden on the patient and caregivers.

## 2. Review Strategy

Evidence for this review was obtained from a search of the Cochrane data base, PUBMED, guidelines of National Institute for Clinical Excellence (NICE), American Academy of Neurology (AAN), and European Federation of Neurological Societies (EFNS); and peer-reviewed journal articles. MND diagnosis is based on the El Escorial diagnostic criteria [4, 5].

## 3. Objectives

This review aims to objectively evaluate the role of the multidisciplinary support care available to patients with MND, the evidence basis for intervention modalities, and highlight areas for future research. The benefit(s) of intervention measures are assessed on their impact on outcome measures such as survival, quality of life (QOL), decreased hospitalization, improved 3 disability, and cost effectiveness.

## 4. Evidence for Multidisciplinary Care (MDC) Strategies and Modalities

### 4.1. Care Setting

MDC approach is the main stay for the management of patients with chronic neurological conditions such as multiple sclerosis [7], stroke [8], acquired brain injury [9], and MND. MDC is defined as any care delivered by two or more disciplines [10], involving a neurologist and other allied disciplines such MND nurse, chest physiologist, and occupational therapist. Other personnel needed as part of the MDC team for MND care includes occupational therapists, physiotherapists, social workers, counselors, speech and language therapist, and religious leaders. Care is administered 24 hours daily in a hospital or on outpatient basis or in the patients' home or community, but effort must be effectively coordinated to avoid overlapping or missing care due to the large number of care providers involved in the management of the patient and their family. MDC is important in enabling care specialist to undertake proper assessment of patients and addressing the concerns of patients and family [11, 12].

An Irish prospective population-based cohort study [13] compared 344 patients in MDC to patients in general neurology care (GNC) and found 7.5-month longer survival in the MDC cohort (*P* < 0.004). Another cross-sectional study involving 208 participants with MND [14] observed an improved QOL in patients with MND who attended MND clinic 6–12 weekly compared to participants who attended a 6-monthly GNC. In a subsequent report [15] observed no difference in healthcare cost between MDC and GNC settings.

In an Italian study involving 126 ALS patients [16], no difference in the median survival time between MDC care and a GNC cohort was reported (17.6 months versus 18 months; *P* = 0.76). The low riluzole and noninvasive ventilation (NIV) use has been suggested as the reason why there was no difference in survival observed in this study [12]. Another Italian study [17], reviewing 221 participants in a MDC setting, noted an improved median survival (*P* = 0.008), decreased hospitalization (1.2 admission frequency versus 3.3, *P* = 0.003), and decreased duration of hospital stay (5.8 versus 12.4 days, *P* = 0.001) in the MDC cohort.

A group [18] retrospectively reviewed hospital notes of 162 patients seen between 1998 and 2002 in GNC and 255 others managed under MDC care between 2006 and 2010 in a tertiary hospital. Median survival from diagnosis was 19 months for MDC and 11 months for GNC (Hazard ratio 0.51, 95% confidence interval 0.41–0.64). They also analyzed the relationship between MDC and survival independent of riluzole, NIV, and PEG use. Although this study selected patients from multiple neurologists in the region, a rigorous methodology was used to ensure proper patient selection and matching. Other factors that are being suggested as contributing to an improved outcome in MDC setting include better symptomatic support, access to aids, and prompt treatment of respiratory challenges [14]. Both AAN and EFNS recommend MDC care setting for patients with MND, with the current EFNS guidelines recognizing the benefit of MDC approach in improving survival, reducing medical complications, and improving the quality of life of patients and their caregivers [19, 20].

### 4.2. Neuroprotective Treatment and Disease-Modifying Therapy (DMT)

The exact molecular pathway leading to the loss of motor neurons in MND remains unclear, but evidence of interplay between complex cellular processes acting synergistically is accumulating [21, 22]. Mutation in super oxide dismutase (SOD1) and TAR-DNA binding protein (TDP-43) among others are strong genetic risk factors [21, 23]. A summary of the genetic and pathophysiologic processes in MND is summarized in Tables 1 and 2.

Riluzole is the only registered DMT for MND which slows the disease progression but does not stop the underlying disorder. The mechanism of action of riluzole involves blocking of presynaptic glutamate release [24]. Four trials [25–28] provided the evidence base for riluzole as a DMT. The first controlled trial involving riluzole [25] reported a modest increase survival among treated patients compared to controls receiving placebo. The same group undertook a subsequent study [26] to address some of the issues raised in the pilot study and confirmed that riluzole is well tolerated and it also extends survival of MND patients. The meta-analysis of these studies showed that irrespective of the patient selection, 100 mg prolongs median survival in people with MND by 2-3 months [29]. Minor reversible adverse reactions reported were nausea, asthenia, fatigue, and an increase in liver enzymes. A recent population-based study [16] found a 6-month overall survival benefit that was significant in bulbar onset and elderly patients, but not in patients with a limb-onset disease. No additional benefits were noted when coadministered with add-on such as Vitamin E [30] or Gabapentin [31].

Recombinant human insulin-like growth factor-1 (rhIGF-1) has been proposed as a MDT in MND following its ability to promote spinal motor neuron survival after excitatory amino acid induced death in animal models [32, 33]. A Cochrane review [34] of the benefit of rhIGF-1 on disease progression using 3 studies involving 799 MND patients observed low quality evidence of improved QOL scores at 9 months, with no impact on survival. A meta-analysis of 2 well randomized trials using ciliary neurotrophic factor (CNTF) as a DMT in 1300 MND patients showed no significant difference between it and placebo, unlike findings reported in animal models which were favorable [35].

The proposed involvement of free radical accumulation and oxidative stress in MND has informed the trial of antioxidants in MND. A meta-analysis of 10 studies involving 1015 MND patients [36] reported weak evidence for the efficacy of antioxidants in MND. Some of the antioxidants which have had positive effect in animal studies are vitamins C and E [37], selegiline [38], N-acetylcysteine [39], and dehydroepiandrosterone [40].

### 4.3. Symptomatic Management

#### 4.3.1. Respiratory Management

Respiratory impairment is the leading cause of death in MND. Denervation weakness of respiratory muscles results in ineffective cough, retention of secretion, and hypoventilation, and it is an important determinant of QOL [41–43]. Its proper management can improve survival and QOL [44]. Onset of respiratory impairment is marked by sleep disordered breathing (SDB) which results in early morning headaches, nonrefreshing sleep, daytime somnolence, dyspnea, orthopnea, poor concentration, and fatigue [41]. Assisted ventilation in MND can be provided using invasive technique via tracheostomy or NIV using face or nasal mask.

In a study involving 22 subjects with MND [45], a 2-monthly assessment of QOL using Short Form Health Survey (SF-36), chronic respiratory disease questionnaire, sleeps apnoea quality of life index, and respiratory function and a 4-monthly polysomnography showed improvement in sleep related problems and mental health that was maintained for 252 to 458 days. Overall survival was significant between NIV patients and those on standard care. Moderate to severe bulbar weakness was associated with a lower improvement in QOL.

In a subsequent randomized control trial (RCT) [46] involving 22 MND patients on NIV and 19 on standard care, a 48-day longer mean survival in the NIV group was observed compared to the standard care group (*P* = 0.0062) at 12 months. In the subgroup with good to moderate bulbar function survival was 205 days longer (*P* = 0.0059). The strength of this study was the computer-based randomized allocation of patients to respiratory support and the even matching of patients and controls in terms of demographic characteristic and functional ability. This study has documented convincingly that NIV prolongs median survival. A review by NICE has further confirmed the cost effectiveness of NIV used for MND patients [44]. NIV improves gas exchange, alleviates symptoms of carbon dioxide retention, and improved QOL [47]. The exact mechanism of action of NIV is unknown, but it may be related to the reversing of chronic respiratory fatigue, reversing of hypercapnia, resolution of atelectasis, and decreasing of the rate of decline of FVC [48]. Claustrophobia, anxiety, excessive salivation, nasal bridge soreness, and abdominal bloating are some of the problems associated with NIV use in MND patients [41].

The effectiveness of NIV can be enhanced by the use of telemonitoring of NIV. Pinto et al. [49] evaluated home telemonitoring of NIV in ALS patients and observed that there was improved survival and lower office and emergency room visit or admissions among patients who had telemonitoring compared with the control group which who had their compliance and ventilator parameter settings assessed only during clinic visits. They concluded that telemonitoring help reduce health care cost and improve survival and functional status.

Invasive ventilation or tracheostomy ventilation (TV) can also be used to deliver air into the lungs and to clear secretions. It is used in patients with severe bulbar dysfunction who cannot tolerate NIV or in patients previously using NIV whose respiratory function has deteriorated to a point where NIV is not tolerated [41]. A retrospective chart review combined with prospective evaluation of QOL and degree of depression [50] obtained a survival rate of 65% by 1 year and 45% by 2 years after tracheostomy. Survival was significantly shorter in patients older than 60 years with a hazard ratio of dying of 2.1 (95% confidence interval, 1.1–3.9). While TV allows for suction of secretion and avoids facemask and claustrophobia, it predisposes to recurrent infection, tracheostomy site infection, bleeding, and tracheaoesophageal fistula formation [51]. It is not encouraged in the USA or Europe but it is the most commonly used respiratory support in Japan [52, 53].

Respiration can also be assisted using an electrical stimulation of the diaphragm to produce contractions (diaphragmatic pacing). It is a procedure originally meant for patients with cervical spine injury [54], but still experimental in MND. Four electrodes are placed on the motor roots of the phrenic nerves on the abdominal surface of the diaphragm. Thus it is only effective if the diaphragm still retains some innervation [41]. The diaphragmatic pacing in patients with respiratory muscle weakness due to motor neurone diseasestudy** (**DIPALS) is an ongoing RCT assessing the efficacy of diaphragmatic pacing among MND patients in some UK hospitals.

Apart from hypoventilation, respiratory weakness also impairs cough [55]. Insufficient cough results in recurrent chest infection, which is the leading cause of hospitalization in MND [56]. The strength of the patients' cough is assessed with a peak flow meter and reported as suboptimal if the peak cough-flow (PCF) is less than 270/min [57]. Cough can be augmented using intensive physiotherapy and manoeuvres like tussive squeeze and mechanical in-exsufflator (MI-E). Evidence for MI-E is weak, but it has been suggested that it could be effective in MND patients for cough management [58, 59].

#### 4.3.2. Nutritional Management

Dysphagia may occur in MND due to loss of coordination, weakness of muscles of mastication, weakness of tongue, and impaired swallowing. This can be complicated by weight loss, distressing choking, prolonged effortful meal times, frequent aspiration, and increase risk of chest infection [60–62]. Malnutrition positively correlates with a shortened survival rate and is a poor prognostic factor [63]. Management of dysphagia at the early stage involves changes to food texture and teaching of swallowing techniques. In the later stages feeding can be through nasogastric tube (NGT) or gastrostomy insertion [64–66]. Current principles of care for dysphagia in MND are based on consensus and expert opinion rather than randomized controlled trials [67].

Guidelines of both the AAN and EFNS recommend the use of gastrostomy in MND [19, 20]. Methods of gastrostomy insertion include percutaneous endoscopic gastrostomy (PEG), radiologically inserted gastrostomy (RIG), and Per-oral image-guided gastrostomy (PIG) [68, 69]. In PEG which is the most commonly used method a wide bore-tube is fixed under endoscopic guidance after sedation to reduce the risk of migration and blockade [70, 71]. The increased risk of aspiration due to sedation and its being unsuitable for patients with moderate to severe respiratory dysfunction are some of the drawbacks of PEG [48, 72].

RIG requires local anaesthesia for its insertion; thus it is observed to have higher success and lower complication rate [68]. It can be used even when FVC is <50%. The disadvantage of RIG is that it is not as securely fixed as PEG and the small tube used can easily be blocked. PIG is a new fluoroscopic technique that has a better long-term clinical outcome in terms of success and complication rates. It requires only local anaesthesia or minimal sedation, and the tube rarely blocks or migrates [70].

There is no evidence in literature regarding which insertion method is superior in any given circumstance [73]. The need for robust randomized prospective trials is encouraged by both AAN and EFNS [19, 64]. Three recent studies [74–76] could not demonstrate any significant difference in survival between PEG and RIG. Another study observed significant difference (*P* = 0.004) between survival in PEG and RIG patients in a subgroup with respiratory failure [68]. The median survival after gastrostomy was 140% higher in the RIG group compared to PEG patients. It is believed that suggestions of PEG being better are just tentative conclusions. [77].

Home parenteral nutrition (HPN) using a central venous catheter is considered as an alternative to long-term nutritional support for MND patients with dysphagia when gastrostomy is contraindicated due to severe respiratory distress. Though it is more expensive than gastrostomy, recent evidence that HPN can be well tolerated and can improve nutritional status is patient with MND [78]. Hypercaloric enteric nutrition has been proposed as a factor that can improve survival because mild obesity is associated with improved survival [79]. Wills et al. [79] evaluated the safety and tolerability of high carbohydrate hypercaloric diet in patients with MND receiving enteric nutrition and observed that when compared to patients receiving isocaloric tube-fed diet or high fat hypercaloric diet, there was lower adverse effect or serious adverse event in the high-carbohydrate hypercaloric group. They were of the view that high carbohydrate hypercaloric enteric nutrition is safe and tolerable in patients with MND.

#### 4.3.3. Other Symptomatic Management

*Sialorrhoea*. Excessive salivation is common in MND as bulbar dysfunction worsens and can be embarrassing or result in aspiration. Amitriptyline, Atropine, Botulin toxin type-B (BTX-B), and external irradiation of the salivary gland have all been tried in the control of sialorrhoea. A double-blind control trial BTX-B injected into the parotid and mandibular gland of 20 patients with refractory sialorrhoea achieved 82% reported improvement compared to 38% among those who received placebo at 2 months (*P* < 0.05) [80]. Costa et al. [81] also evaluated the efficacy and safety of BTX-B in the treatment of sialorrhoea in patients with a bulbar onset MND in an open-labeled prospective study that involved the injection of BTX-B into the parotid and submandibular glands. They observed that most patients reported a better quality of life while on treatment and a mean reduction of symptom severity of 70%. The most commonly reported side effects include viscous saliva, local pain, chewing weakness, and respiratory infection.

*Bronchial Secretion*. Bulbar impairment results in poor clearance of tenacious sputum. Mucus accumulation is a poor prognostic factor in patients on NIV [82]. No RCT exists for any of the treatment approaches in MND. EFNS recommends the use of mucolytics such as N-acetylcysteine when there is sufficient cough flow. 

*Pseudobulbar Affect*. This is observed in up to 50% of MND patients [83]. Yawning, weeping, and laughing are the characteristic presentation. Pseudobulbar affect has a negative impact on QOL. Small placebo controlled trials and case series have observed the effectiveness of selective serotonin reuptake inhibitors (SSRI) and tricyclic antidepressants in controlling this symptom [84, 85]. Pioro et al. [86] evaluated the treatment of pseudobulbar affect in patients with multiple sclerosis and MND in a randomized-controlled trial using 30/10 mg and 20/10 mg Dextromethorphan plus ultra-low-dose quinidine. They reported that dextromethorphan plus ultra-low-dose quinidine is effective in reducing the frequency and severity of symptoms and improving patients' quality of life especially at the dose of 30/10 mg combination when compared with placebo. EFNS recommends Citalopram (SSRI) and Amitriptyline (TCA) for treatment of troublesome cases of pseudobulbar affect [20]. A fixed dose combination of dextromethorphan/quinidine (30 mg/30 mg) is the recommended treatment option of AAN [87]. 

*Cramps*. It is usually troublesome particularly at night. A RCT failed to support the efficacy of tetracannabinoid in treating moderate to severe cramp [88]. A small open-labeled pilot study confirmed that levetiracetam is useful for treatment of cramps in MND patients [89]. Modalities such as massage, physical exercise, hydrotherapy, heated pools, and drugs like carbamazepine, diazepam, phenytoin, and verapamil have all been tried without any conclusive evidence. EFNS recommends levetiracetam, quinine sulfate, and physical therapies for management of cramp in MND. 

*Spasticity*. Physical therapy is the main treatment modality for spasticity that has its usefulness established from randomized controlled trial in literature [20, 90]. Physical therapy methods in use include therapeutic exercise, stretching, positioning, casting, and biofeedback. Other interventions with no controlled trial evidence include heat/cold therapy, hydrotherapy, ultrasound, electrical stimulation, and chemodenervation and rarely surgery can be used [20]. Intrathecal Baclofen is the drug of choice in intractable cases [20, 91]. Drugs such as Dantrolene, Tetrazepam, and Tizanidine have not been tested in MND in clinical practice but are recommended by EFNS. Nonpharmacological treatment modalities should first be deployed before pharmacological interventions are introduced if symptoms do not improve. These drugs should be used with caution in MND patients as they can cause depression of respiration and worsening of weakness. Physical therapy can be combined with one or more of the antispasticity drugs [92]. 

*Insomnia and Fatigue*. Insomnia is common in the final stages of MND probably due to cramps, pain, and respiratory impairment [93]. Amitriptyline and Zolpidem are some of the medications used in practice without being tested. Fatigue is potentially debilitating and may be of central or peripheral origin. An open-labeled trial confirmed the effectiveness of Modafinil in the treatment of fatigue in MND [94, 95]. 

*Cognitive Impairment*. MND is associated with a frontotemporal type of dementia and it is associated with a negative impact on survival [96]. Cognitive impairment has been demonstrated in 20–50% of patients with MND. A number of screening instruments are available for assessing cognitive impairment in MND, but EFNS recommends the use of tools that can assesses verbal fluency as a major component of any test instrument [20]. 

*Communication*. It is important for effective social interactions. Subtle changes may be seen as word finding difficulties, spelling difficulty, and decreased verbal output. Language impairment results in difficulties in clinical management and decreases QOL of patients and caregivers [97]. EFNS recommends 3–6 monthly assessment; full neuropsychology test; and use of communication aids like computerized speech synthesizer [20]. 

*Palliative Care*. This is a holistic management of patients with terminal disease aimed at optimizing the QOL of patients and their caregivers. Palliative care in MND should ideally start at the point of diagnosis and continue throughout the entire history of the disease. It is needed at the different stages of the disease such as at the time of diagnosis to address issues relating to breaking of the bad news to patient and their caregivers; during crises points for example, introduction of NIV or PEG; and terminal stage when the patients' condition deteriorates. The application of the palliative care techniques should be organized to meet the need of individual patients as the reaction of patients and their families to the evolution of the disease varies between patients. It should focus on aspects of care such as physical (symptom control); psychological (effect of disease on patients); social (impact of disease on family and care-givers); and spiritual (questions bordering on meaning of life and the fear of dying). The approach should integrate both clinic and community-based care from onset of disease and continue even after the patient has died [11, 98]. EFNS guidelines [20] recommend early palliative care referral with discussion covering aspects of end-of-life, advance directive, and naming of health-care proxy.

Steinhansen et al. [99] evaluated a group of seriously ill patients, recently bereaved patient relatives, physicians, and other healthcare providers in a study to determine the factors considered to be important at end-of-life. All respondents agreed that naming a decision maker, maintaining one's dignity, having a care provider one can trust, to be pain free, having one's financial affairs in order, be free of shortness of breath and anxiety, to have a physician who is comfortable talking about death and knowing that one's family is prepared for their death among others are issues which are important for palliative care. They also largely accepted pain and symptom management as issues that palliative care should address.

## 5. Areas Needing Further Research in MND

Further research is needed in examining referral bias to MDC and other factors, which may influence effectiveness of MDC clinic outcomes such as frequency of visit. Biomarker for early diagnosis should be evaluated as this aids in research into DMT. Better evidence is still needed in the best parenteral feeding modality and the most optimal time of initiating/withdrawing it; impact of cough-assisting devices; nutritional management techniques on the QOL; evaluation of language dysfunction and its management treatment; the cost effectiveness of different treatment modalities; approach to the management of terminal care; and evidence for advance directives, impact of disease, and effect of disease on QOL of caregivers.

## 6. Conclusion

MND is a fatal syndrome with short disease course and low incidence. Scarcity of evidence for most of current treatment modalities requires further trials to standardize care for patients.

## Figures and Tables

**Table 1:** Genetics of MND.

Type of MND	Genetic mutation
Familial MND	(i) SOD1 gene [100].
	(ii) TDP-43 [101–103].
	(iii) Alsin (ALS2) [104, 105].
	(iv) Senataxin (ALS4) [106].
	(v) Vesicle associated membrane-protein (VAPB, ALS8) [107].
	(vi) Angiogenin [108, 109].
	(vii) Mutation in the p150 subunit of dynactin (DCTN1) [110, 111].

Sporadic MND	Genetic mutations linked to greater susceptibility to sporadic MND include
	(1) apolipoprotein E4, [112]
	(2) decreased expression of excitatory amino acid transporter-2 protein [113, 114],
	(3) alterations in the vascular endothelial growth factor (VEGF) gene [115].

**Table 2:** Pathophysiological processes in MND.

Pathophysiologic process	Comments
Excitotoxicity	Excessive postsynaptic glutamate induced stimulation of glutamate receptors such as NMDA & AMPA → massive calcium influx → nitric acid formation and neuronal death [21, 116].

Oxidative stress	Fibroblast culture from MND patients shows increased sensitivity to oxidative damage. Accumulation of free oxygen species → cell death. SOD1 is an antioxidant enzyme [116].

Mitochondrial defect	Abnormalities of mitochondrial morphology and biochemistry have been reported in sporadic MND patients, in SOD1 transgenic mice, and in cellular models [117, 118].

Impaired axonal transport	The relatively long length of motor neuron depends on effective transport systems. Evidence of abnormalities in this transport system has been reported in transgenic mice [119–121].

Neurofilament aggregation	Abnormal accumulation of neurofilament commonly occurs in many neurodegenerative diseases including MND [122, 123].

Protein aggregation	Intracellular inclusions have been observed in MND. The evidence is still unclear if these proteins are toxic or beneficial to the cell [21, 22].

Inflammatory dysfunction	Evidence suggests the possibility of an inflammatory process [23].

Deficits in neurotrophic factors and dysfunction of signaling pathway	Deficits in levels of neurotrophic factors, e.g., IGF-1, have been reported in MND [124–126].

Apoptosis	The final process in MND leading to neuronal death is said to closely resemble apoptosis, and markers of apoptosis have been detected in the later stages of the disease and animal models [127–129].
